# Dental fear in school children and young adults attending public dental health care: prevalence and relationship to gender, oral disease and dental treatment; trends over 40 years

**DOI:** 10.1186/s12903-022-02166-6

**Published:** 2022-04-26

**Authors:** Anna Nydell Helkimo, Bo Rolander, Göran Koch

**Affiliations:** 1 Department of Paediatric Dentistry, The Institute for Postgraduate Dental Education, Region Jönköping County, Box 1030, 551 11 Jönköping, Sweden; 2 Futurum, Academy for Health and Care, Region Jönköping County, Jönköping, Sweden; 3 Department of Behavioral Science and Social Work, School of Health Sciences, Jönköping University, Jönköping, Sweden

**Keywords:** Dental fear, School children, Young adults, Oral health, Dental treatment, Public dental health care

## Abstract

**Purpose:**

To study prevalence of dental fear and the relationship to gender, oral disease and dental treatment between 1973 and 2013 in school children and young adults attending public dental health care.

**Methods:**

Every ten years from 1973 to 2013 random samples of about 100 individuals in each of the age groups 10, 15 and 20 years took part in a repeated cross-sectional study based on clinical parameters and a questionnaire. Dental fear was estimated by the question: *“What do you feel at the prospect of an appointment with a dentist?”.* 75–99% of the samples answered the question. Agreement to at least one of the alternative answers: *ill at ease, frightened* and *sick* defined *dental fear. Frightened* and/or *sick* indicated *severe dental fear.* The prevalence of caries, gingivitis and number of filled tooth surfaces were calculated. Chi-square tests were used to show differences in proportions between groups and linear regression to show trends over time.

**Results:**

Prevalence of *dental fear* declined in all age groups over time. In the 20-year olds *dental fear* was found in 29% of the sample and *severe dental fear* in 12% of girls and 5% of boys in 2013. Individuals with *dental fear* had higher mean caries prevalence and number of filled tooth surfaces compared with individuals without dental fear.

**Conclusions:**

This 40-year time trend study showed a reduction in dental fear prevalence in school children and young adults offered regular public dental health care based on prevention and a psychological approach. The prevalence of dental fear was still high in 2013 despite a significant decline in caries during the study period. Further improvements in the psychological approach when treating children are thus needed.

## Introduction

Oral health promotion directed to the entire child population is the overall objective for a public dental health care system. According to the United Nations Convention on the Rights of the Child, every child has the right to attain good oral health [1]. Oral health is a broad concept embracing both absence of oral disease and dental fear [2]. Therefore, prevention of oral disease as well as dental fear should be the priority for dental teams treating children.

Dental fear is common in children [3–5]. The prevalence of dental fear has been investigated in cross-sectional studies since the 1970s [4, 6]. The reported prevalence varies widely, though has been estimated to be 9% in children and more prevalent in girls compared with boys [5]. Longitudinal studies of dental fear prevalence are few [7–10]. The relation between age of the child and dental fear prevalence is not clear; both decrease and increase in dental fear prevalence in older ages is reported [5]. In addition to longitudinal studies, repeated cross-sectional studies of a population show changes in prevalence and trends over time. A few repeated cross-sectional investigations of dental fear in children offered public dental health care have been published [11, 12]. These time trend studies give some support for the hypothesis that a public dental health care programme results in less dental fear in the child population. Regular dental visits from an early age, a psychological approach and introduction to dental treatment, caries prevention and pain control are factors included in child dental health care programmes and are of importance for dental fear prevention [10, 13, 14].

A 40-year time trend study of dental attitudes in a child population offered a comprehensive public dental health care programme in Jönköping, Sweden, showed a shift from negative to more positive dental attitudes [15]. Dental fear is one aspect of negative dental attitudes [16]. Pain and a feeling of losing control during dental treatments are strong causal factors of dental fear [5]. A greater effect on dental attitudes and reduction of dental fear prevalence was expected, since a dramatic decline in caries prevalence and need of restorative dental treatment had occurred during the same 40-year period [17]. This limited outcome of dental fear reduction most likely mirrors the multifactorial aetiology and multidimensional character of dental fear [5, 18].

Published time trend studies on dental fear in school children offered public dental health care have focused on single age groups [11, 12]. A time trend study of dental fear in populations of school children and in young adults just leaving the public dental health care system would provide information not only of trends in dental fear prevalence, but also the end result of a public dental health care system offered throughout childhood.

The aim of this 40-year time trend study is twofold: to analyse changes over time of dental fear prevalence in populations of school children and young adults offered public dental health care and to study the relationship between dental fear and gender, oral disease and restorative dental treatment.

## Materials and methods

Random samples of 10-, 15- and 20-year-olds were invited to a series of repeated cross-sectional epidemiological studies (*the Jönköping studies)*, in 1973, 1983, 1993, 2003 and 2013. Information about the sampling process and ethical approval (Ethic Committee at the University of Linköping, Linköping, Sweden. Dnr 2012-191-31) and dropouts has been provided in a previous paper [19]. After written consent, about 100 individuals of each age group were included in each study year. The parents consented on behalf of the 10- and 15-year-olds. In 2013, written consent was also provided by the children 15 years of age. *The Jönköping study* includes a comprehensive questionnaire and a clinical and radiographic examination performed in a dental setting [19, 20]. No operative dental treatment was offered. The participants were free to participate in the entire study or merely part thereof.

The investigation in 1973 was a baseline oral health description at the time for implementation in Sweden of an extended public dental health care system, including the entire child population between 0 and 19 years of age (Dental Act, SFS 1973:457). Since 1974, the child population in the County of Jönköping, Sweden, has been offered a comprehensive programme for oral health promotion and disease prevention [21]. The preventive programmes were both general i.e. directed to the whole child population irrespective of risk and individualized. The programmes focused on oral hygiene, dietary advice and fluoride use. The responsibility of the individuals was stressed. The tell-show-do method for a stepwise introduction and a psychological approach when treating children were also implemented [22]. Improvements to the preventive programmes were made continuously. Oral health status at the age of 20 mirrors the end result of a public dental health care programme for children between 0 and 19 years of age.

A prerequisite for the comparison of results from repeated studies is that no changes are made to the sampling process, study design, definitions and clinical criteria during the study period. However, due to the decline in caries prevalence, the number of radiographs was reduced. Radiographs recently undertaken at a dental clinic as part of an individual regular examination were available. Information about clinical findings and radiographs from the study examination were given to the dentist responsible for the regular dental treatment.

To mirror dental attitudes, the question: *“What do you feel at the prospect of an appointment with a dentist?”* was used. The question and alternative answers were a modification of the questions and answers included in the Dental Anxiety Scale (DAS) developed by Corah [23]. Parents assisted the 10-year-olds in filling out the questionnaire. Five alternative answers were possible and more than one could be chosen. The alternative answers were: *full of expectation, unaffected, ill at ease, frightened* and *sick.* The inclusion criterion for the present study was an affirmative answer to at least one of the five statements. Individuals who did not agree with any of the proposed answers were excluded. The number of 10-, 15- and 20-year-olds in the study group and the percentage of the original age groups in *the Jönköping studies* are presented in Table 1. For one of the 20- year old participants in 2013 sex was not registered.

**Table 1:** Number of individuals in 10-, 15-, and 20-year-old groups who fulfilled the inclusion criteria (study group) and percentage of the total number of individuals in the original studies in 1973, 1983, 1993, 2003 and 2013

Age group	Number examined																			
	1973				1983				1993				2003				2013			
	Female	Male	n	%	Female	Male	n	%	Female	Male	n	%	Female	Male	n	%	Female	Male	n	%
10	45	47	92	92	42	63	105	95	41	61	102	89	43	44	87	75	33	54	87	92
15	45	37	82	82	51	44	95	89	48	40	88	86	40	34	74	77	44	40	84	83
20	59	37	96	96	55	44	99	99	45	47	92	92	34	39	73	87	48	21	70	93

The definition of dental fear was agreement to at least one of the three statements *ill at ease*, *frightened* and *sick.* Individuals who answered *full of expectation* and/or *unaffected* were considered to have *no dental fear.* The study group was divided into a *dental fear group* and a *no dental fear group.* A subgroup within the *dental fear group,* called the *severe dental fear group*, included those individuals who answered *frightened* and/or *sick.* The prevalence of *dental fear* and *severe dental fear* in each age group and in girls and boys respectively, were calculated in all study years.

Ten-, 15- and 20-year-olds with *no dental fear* and *dental fear* respectively, were compared concerning the prevalence of caries and gingivitis and filled tooth surfaces. Only permanent teeth were analysed. Caries prevalence, including both initial and manifest lesions, was registered clinically and radiographically. The DFS index (Decayed Filled Surface index) was used to describe the tooth surfaces (S) with caries lesions (D) and fillings (F). In a child population included in a regular public dental health care system tooth surfaces with manifest carious lesions will be restored. Therefore, D in the index mainly indicate initial carious lesions. The DFS index is derived from the DMF system presented in 1938 [24]. In the studied populations no permanent tooth had been extracted due to dental decay [20] and therefore missed surfaces (M) were excluded from the index used in this study. The number of filled tooth surfaces of permanent teeth (FS) was extracted from the DFS index. Gingivitis was defined as gingival tissue bleeding on gentle probing. Four sites on each tooth were examined. The number of sites with bleeding as a percentage of the total number of tooth sites available was calculated.

### Statistical analysis

Descriptive statistics were used to present the results. The prevalence of *dental fear* and *severe dental fear* in 10-, 15- and 20-year-olds respectively, was presented as a percentage of the study groups in each study year. To compare the prevalence of *dental fear* and *severe dental fear* in 10-, 15- and 20-year-olds, Chi-square tests were used. The prevalence of *dental fear* and *severe dental fear* in girls and boys respectively, at the ages of 10, 15 and 20, was presented as a percentage of the number of girls and boys in the study groups in each study year. Further, to compare prevalence of *dental fear* and *severe dental* fear in girls and boys, Chi-square tests were performed. To find possible trends in *dental fear* and *severe dental fear* prevalence in 10-, 15- and 20-year-olds over the 40 years, a scatter plot was constructed and a linear regression line was calculated.

The mean numerical values for DFS and FS, and concerning gingivitis, the mean percentage of tooth sites with bleeding, were calculated for the *no dental fear* and *dental fear groups* of 10-, 15- and 20-year-olds at the five study years. Mean values of DFS, FS and gingivitis percentage were used to mirror the collective burden of oral disease and treatment need in the different groups. Prevalence of dental disease showed a skewed distribution according to the Shapiro–Wilks and Kolmogorov–Smirnov normality tests. Therefore no statistical analysis of differences in mean values of dental parameters in the *no dental fear* and *dental fear groups* was performed. Due to the small number of individuals included in the study, multivariate analysis of dental fear and related factors could not be used. The data processing was performed using SPSS version 25 (IBM Corporation, Armonk, New York State, USA). The significance level was set at p < 0.05.

## Results

### Prevalence of dental fear

**Fig. 1:**
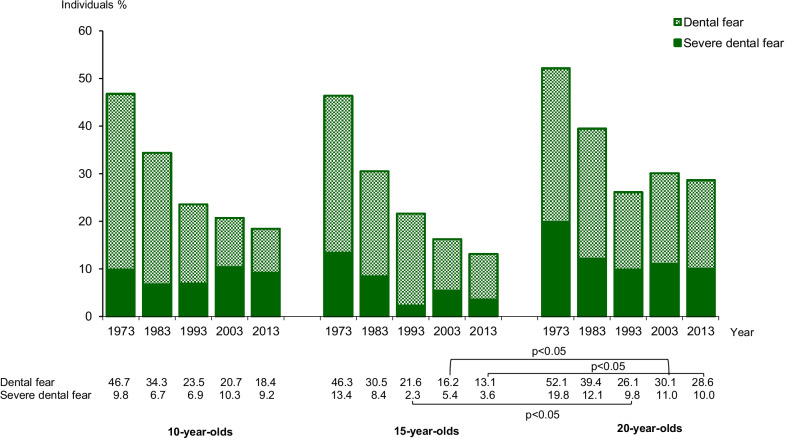
Percentage of 10-, 15-, and 20-year-olds with dental fear and severe dental fear in 1973, 1983, 1993, 2003 and 2013. Severe dental fear group is included in dental fear group

Figure 1 illustrates the prevalence of *dental fear* and *severe dental fear* as a percentage of 10-, 15- and 20-year-olds. During the 40-year study period, the prevalence of *dental fear* declined in all age groups. The main reduction in dental fear in the 20-year-olds was seen during the first 20 study years. In 2013, *dental fear* was twice as prevalent in the 20-year age group compared with the 15-year age group. Over the study period *severe dental fear* prevalence was fairly constant in 10- and 20-year-olds, but declined in 15-year-olds.

**Fig. 2:**
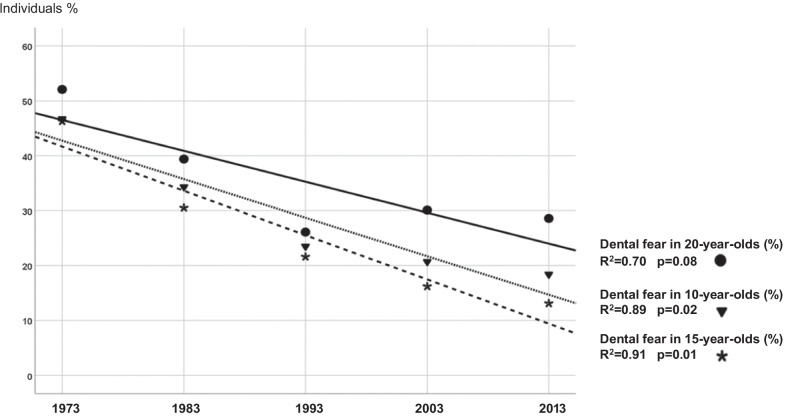
Trends in dental fear prevalence in percentage of 10-, 15- and 20-year-olds during 40 years (1973–2013)

Figure 2 shows the *dental fear* regression lines indicating a decreasing trend in *dental fear* prevalence in all age groups over the 40-year study period. No statistically significant trend in *severe dental fear* prevalence was seen.

**Fig. 3:**
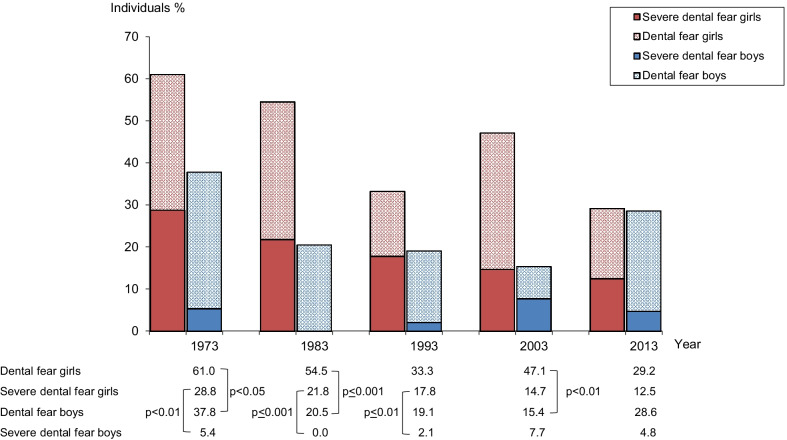
Percentage of 20-year-old girls and boys, respectively, with dental fear and severe dental fear in 1973, 1983, 1993, 2003 and 2013. Severe dental fear group is included in dental fear group

Figure 3 shows the prevalence of *dental fear* and *severe dental fear* in girls and boys respectively, at the age of 20 and over the 40-year period. In all study years except 2013, higher prevalence figures of *dental fear* and *severe dental fear* were registered in girls compared with boys. In 2013, the prevalence of *severe dental fear* was 12% in girls and 5% in boys.

In 15-year-old girls, *dental fear* and *severe dental fear* were more prevalent compared with boys. The difference in *dental fear* was statistically significant in 1983 (*p* < 0.05) and 1993 (*p* < 0.01). Ten percent of the 15-year-old girls and none of the boys reported *severe dental fear* in 2003. At the age of 10 no consistent difference in *dental fear* and *severe dental fear* prevalence in girls compared with boys was seen during the study period.

### Dental fear and caries prevalence

**Fig. 4:**
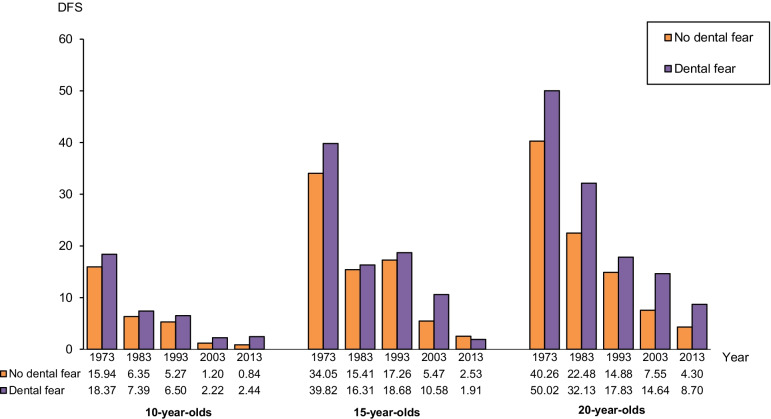
Mean number of DFS in 10-, 15-, and 20-year-olds with no dental fear and dental fear, respectively, in 1973, 1983, 1993, 2003 and 2013

Figure 4 shows the reduction in caries prevalence during the study period in *no dental fear* and *dental fear groups* of all three age groups. Higher mean caries prevalence (DFS) was registered in the *dental fear groups* compared with the *no dental fear groups,* with a single exception for 15-year-olds in 2013. Caries-free individuals (DFS 0) constituted an increasing part of the age groups and was in 1973–2003 more prevalent in the *no dental fear groups* compared with *dental fear groups*. Table 2 shows the percentage of 10-, 15- and 20-year olds, subdivided into groups with and without dental fear, with DFS 0.

**Table 2:** Percentage of individuals with DFS 0 at the age of 10, 15, and 20 years, respectively, subdivided into individuals with no dental fear and dental fear in 1973, 1983, 1993, 2003, and 2013

Year	10 years		15 years		20 years	
	No dental fear	Dental fear	No dental fear	Dental fear	No dental fear	Dental fear
1973	4.1	0	0	0	0	0
1983	4.3	0	3.0	3.4	1.7	0
1993	6.4	4.2	2.9	0	4.4	0
2003	59.4	44.4	21.0	16.7	17.6	4.5
2013	62.0	50.0	46.6	54.5	20.0	20.0

### Dental fear and gingivitis prevalence

**Fig. 5:**
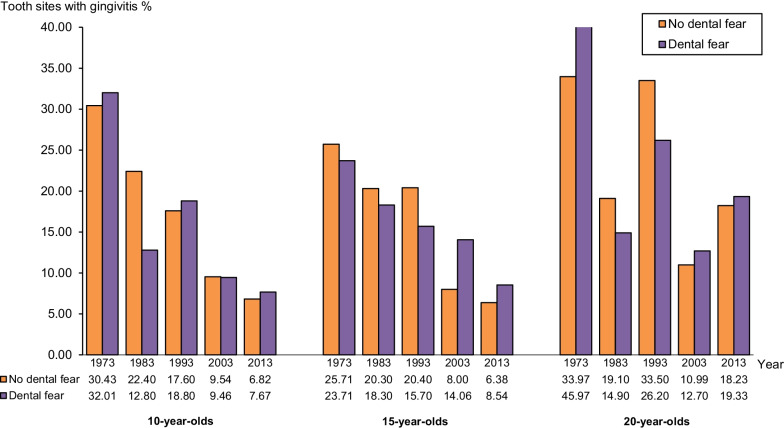
Mean percentage of tooth sites with gingivitis in 10-, 15- and 20-year-olds with no dental fear and dental fear, respectively, in 1973, 1983, 1993, 2003, and 2013

Figure 5 shows the mean values of gingivitis in the *dental fear* and *no dental fear groups* of 10-, 15- and 20-year-olds. The prevalence of gingivitis in each age group and year of examination were with some exceptions, almost equal in groups with and without dental fear. From 1973 to 2013 a reduction in the mean percentage of tooth sites with gingivitis was seen. The lowest mean values (6–9%) were registered 2013 in *groups* of 10- and 15-year-olds. In the groups of 20-year-olds, bleeding was observed at approximately 20% of available tooth sites in 2013.

### Dental fear and filled tooth surfaces

**Fig. 6:**
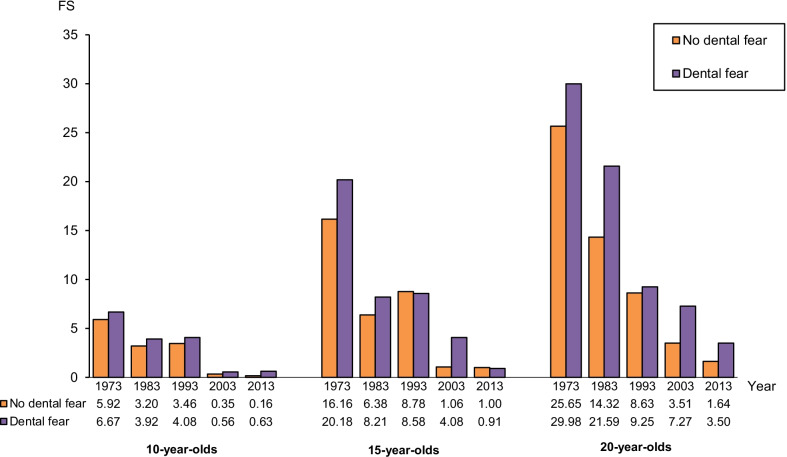
Mean number of filled tooth surfaces in 10-, 15-, and 20-year-olds with no dental fear and dental fear, respectively, in 1973, 1983, 1993, 2003 and 2013

Figure 6 shows the mean number of filled tooth surfaces (FS) in *dental fear* and *no dental fear groups* in all age groups and study years. A reduction in filled tooth surfaces was seen in all groups, but in general, d*ental fear groups* had higher mean FS values compared with *no dental fear groups*. The mean number of filled tooth surfaces were low in all age groups in 2013.

## Discussion

The present time trend study over an extended time period of 40 years shows a decrease in dental fear prevalence in school children and young adults included in a regular public dental health care system based on prevention and a psychological approach. The study also shows that oral health was improved in the studied populations, though the improvements were greater in groups without dental fear compared with those with dental fear. Since a lower number of restorations were performed in the child population, the risk of negative treatment experiences was reduced during the study period. This fact and the step wise introduction and psychological approach used when treating children, are most probably the reasons for the reduction in dental fear prevalence during the study period. The present study thus suggests that a regular public dental health care leads to a reduction in dental fear prevalence and is in line with results from previous comparable time trend studies [11, 12].

Even if a statistically significant decreasing trend of *dental fear* prevalence in 10- and 15-year-olds and a 45% reduction in dental fear prevalence in 20-year-olds were registered from 1973 to 2013, the prevalence of *dental fear* was still high in 2013. Prevalence of *severe dental fear* showed only minor changes during the studied period. This is in line with a two year longitudinal study of dental fear in 15- to 21-year olds in Norway. The dental fear prevalence declined, while the group with severe dental fear was constant [10]. General anxiety and depression are factors strongly related to dental fear in adolescents [25]. Temperament with activity and impulsivity is also correlated to dental fear prevalence [26]. The limited effect of a public dental health care system on severe dental fear prevalence might be explained by mental health and personality factors.

No reduction in dental fear prevalence was seen in the 20-year-olds from 1993 to 2013, despite a continuous caries decline and lower number of tooth fillings. However, 20-year olds with good oral health, might have had earlier negative experiences related to caries treatment of primary teeth. Dental fear often emerges during childhood [27]. Caries development at an early age is the strongest risk factor for further caries development [28] and restorative treatment of primary teeth often requires revision [29]. Caries prevention in the primary dentition will most probably reduce the risk of persistent dental fear in young adults.

Young adults with dental fear at the termination of the public dental health care system form a risk group, since dental fear seems to be more persistent in adults compared to children [9, 12, 27, 30]. Dental fear is an oral health problem affecting the individual, the dental team and society [4, 31, 32]. Dental fear can lead to avoidance and no attendance to dental care as well as risk of deteriorated oral health [12, 14]. On the other hand, a reduction in dental fear leads to more regular dental attendance [33]. Against this background, more efforts should be directed to prevent and treat dental fear during childhood.

The fact that a routine oral examination might cause discomfort and even pain must be considered [12]. Pain and feelings of losing control during treatment should always be avoided [5]. Dental care for children should be based on a psychological approach including information and communication, stepwise introduction, time to adjust to the dental setting and pain control [5, 10, 12, 34]. A trusting relation between child patients and the dental team is important and can be established if the child’s opinion is respected and the child is involved in treatment planning. This is in line with the UN Convention on the Rights of the Child, passed as a Swedish law in 2020 [1].

Results from the present study indicate a relationship between dental fear and age and sex respectively. In 2013, the subdivision *severe dental fear group* constituted half of the *dental fear group* at the age of 10 and one third at the ages of 15 and 20. Thus younger school children with dental fear might have more pronounced dental fear compared with older school children. A female predominance in dental fear prevalence was found in the present study at the age of 15 and 20, which is in accordance with several other studies [3, 9, 12, 18, 30, 35]. The lowest prevalence of *severe dental fear* was in the present study registered in 15-year-olds, especially among boys. Social norms and expectations might explain these gender differences [35].

The prevalence of dental fear in 2013 at the age of 20 was 29%—much higher than reported in a time trend study from Norway, where dental fear was found in 8% of 18-year-olds when leaving the public dental health care system [12]. The prevalence of dental fear in Norwegian young adults is comparable with the prevalence of *severe dental fear* at the age of 20 reported in the present study. Different methods and different dental fear definitions and criteria might explain the diverging results, since the public dental health care systems in the two countries are similar.

The present study showed, especially at the age of 20, a positive relation between dental fear and caries prevalence and filled tooth surfaces. Caries lesions and restorations at the age of 20 indicate further need of dental restorative treatment as an adult. No difference in gingivitis prevalence related to dental fear was found. Dental fear might lead to improved oral hygiene routines to prevent future dental treatment need. However, bleeding at 20% of available tooth sites at the age of 20 in 2013 indicates a considerable need of improved oral hygiene.

The present investigation is based on repeated clinical examinations and questionnaires during 40 years. This is the major strength of the study. To execute such studies demands, not only large personal resources, but also skills in planning and performance. No alterations in the sampling process, methods, clinical definitions and criteria would be made if trends and relationships between dental fear and clinical parameters are to be compared over time. Thus, the same study design, set in 1973, had to be followed in the five repeated examinations.

It can be questioned if a single question could give good information about dental fear. However, it has been stated, that a single question to mirror dental fear is reasonable and acceptable in extensive questionnaires on oral health [36]. This method is used in several other epidemiological studies and has been found to be reliable, at least in adults [37]. The validity and reliability of the single question and the proposed answers used in *the Jönköping study* was not evaluated in the present study. The question and answers were a modification of the questions with proposed answers used in the Dental Anxiety Scale (DAS) developed by Corah [23]. The DAS system was the dental fear scale used in the 1970s and has been evaluated and found suitable to measure dental fear in clinical practice and research projects [38]. The questionnaire was filled out in connection to the dental examination. The dental setting might have had a negative impact on reports concerning dental fear [39]. Dental fear in parents might have influenced the answers of the 10-year-olds, as parents helped the child in filling out the questionnaire. Parents with dental fear are more likely to assess dental fear in their children compared with parents without dental fear [40].

## Conclusions

This 40-year time trend study shows a reduction in dental fear prevalence in populations of school children and young adults offered a regular public dental health care programme based on prevention and a psychological approach. Despite a dramatic caries decline and less need of restorative treatments, dental fear is still common 40 years after implementation of the programme. Thus, improvements in the psychological approach are necessary to further reduce dental fear in children. Since improvements in oral health—though to a lesser degree—were seen in individuals with dental fear, dental fear is a caries risk indicator in child populations with a generally low caries prevalence. Severe dental fear in teenagers and young adults was mainly found in girls. Individuals with *severe dental fear* should be identified early, as they are prone to avoid dental visits and also show a greater treatment need.

## Data Availability

The datasets used and/or analysed during the current study are available from the corresponding author on reasonable request.
